# Protective effect of calcitriol on rhabdomyolysis-induced acute kidney injury in rats

**DOI:** 10.1038/s41598-019-43564-1

**Published:** 2019-05-08

**Authors:** Natany Garcia Reis, Heloísa Della Coletta Francescato, Lucas Ferreira de Almeida, Cleonice Giovanini Alves da Silva, Roberto Silva Costa, Terezila Machado Coimbra

**Affiliations:** 1 Departments of Physiology, University of São Paulo, Ribeirão Preto, São Paulo Brazil; 2 Departments of Pathology of Ribeirão Preto Medical School, University of São Paulo, Ribeirão Preto, São Paulo Brazil

**Keywords:** Physiology, Nephrons

## Abstract

Glycerol injection in rats can lead to rhabdomyolysis, with the release of the intracellular muscle content to the extracellular compartment and acute kidney injury (AKI). Oxidative stress and the inflammatory processes contribute to the disturbances in renal function and structure observed in this model. This study evaluated the effect of calcitriol administration in AKI induced by rhabdomyolysis and its relationship with oxidative damage and inflammatory process. Male Wistar Hannover rats were treated with calcitriol (6 ng/day) or vehicle (0.9% NaCl) for 7 days and were injected with 50% glycerol or saline 3 days after the beginning of calcitriol or saline administration. Four days after glycerol or saline injection, urine, plasma and renal tissue samples were collected for renal function and structural analysis. The oxidative stress and the inflammatory processes were also evaluated. Glycerol-injected rats presented increased sodium fractional excretion and decreased glomerular filtration rates. These alterations were associated with tubular injury in the renal cortex. These animals also presented increased oxidative damage, apoptosis, inflammation, higher urinary excretion of vitamin D-binding protein and decreased cubilin expression in renal tissue. All these alterations were less intense in calcitriol-treated animals. This effect was associated with decreases in oxidative damage and inflammation.

## Introduction

Rhabdomyolysis is characterized by skeletal muscle injury. It is usually associated with trauma, but it also occurs in several clinical conditions, including abrupt changes in body temperature, strenuous physical exercise, prolonged muscle compression, exposure to toxins and drugs and infections^1,2^. Acute kidney injury (AKI) is the most serious complication; it is estimated that 10% to 40% of patients with rhabdomyolysis develop AKI^3–5^. The mortality rate is approximately 20% in patients who do not develop kidney damage^6^ and increases to 59% when AKI is present^7^. Glycerol-induced AKI is an animal model used to understand the clinical syndrome and the general mechanisms of renal lesions^6,8^. The injury of more than 100 grams of skeletal muscle exceeds plasma protein binding capacity for myoglobin, leading to glomerular filtration and higher reabsorption of myoglobin by the cells of the proximal renal tubules with precipitation of free myoglobin in the renal distal tubules, provoking tubule necrosis and obstruction with loss of renal function and renal structural damage^9^. Renal vasoconstriction is a characteristic feature of the rhabdomyolysis-induced AKI. The activation of the renin-angiotensin system, vasopressin and the sympathetic nervous system occurs after intravascular volume depletion provoked by extracellular fluid sequestration due to the muscle lesion^1^. The increased levels of endothelin-1, isoprostanes and other vascular mediators also contribute to the reduction of renal blood flow^10,11^. The oxidative damage is present, with decrease of antioxidant enzymes (superoxide dismutase; SOD)^12^ and increase of oxidative markers (isoprostanes and nitrotyrosine)^11,13^ leading to the activation of proinflammatory pathways, including nuclear factor-κB (NF-κB)^13^ and c-jun N-terminal kinase (JNK)^14^, contributing to the necrosis of tubular epithelial cells.

The primary physiological actions of the biologically active metabolite of vitamin D, 1,25-dihydroxyvitamin D3 (1,25 (OH)_2_D_3_), also called calcitriol, are calcium and phosphorus uptake and transport, controlling bone formation^15^. However, over the last several years, studies have shown the importance of calcitriol in other systems as well, including cell proliferation and differentiation^16–18^, as well as inflammatory processes. Recent studies have shown that calcitriol protects the kidney by targeting JNK^19^ and the NF-kB pathways^20^, decreasing the production of pro-fibrotic and proinflammatory factors and oxidative stress. Sun *et al*.^21^ observed that pretreatment with calcitriol significantly reduced isoprostane expression in placental trophoblast cells in response to hypoxic stimulation. In addition, 25-hydroxyvitamin D3, the inactive form vitamin D, carried by the vitamin D binding protein (VDBP) is reabsorbed via the cubilin receptor and is activated in proximal tubule cells^22^. Therefore, lesions of this segment reduce renal reabsorption and activation of vitamin D.

This study evaluated the effect of treatment with calcitriol on renal structural and functional alterations caused by the administration of glycerol in rats and relationship of such treatment with oxidative damage and the renal inflammatory process.

## Results

### Renal function and plasma creatinine kinase and calcium levels

The animals injected with glycerol showed higher sodium fractional excretion (FENa^+^), increased plasma creatinine (Cr), plasma creatine kinase (CK) and lower glomerular filtration rate (GFR) than did the control groups, indicating decreased renal function and muscle injury. In addition, increased urinary flow and decreased urinary osmolality (Uosm) were observed in these animals. All these changes, except the urinary osmolality and plasma creatinine, were less intense in animals treated with glycerol + calcitriol. There was no difference in plasma calcium between groups, 96 hours of glycerol injection (Table 1).

**Table 1:** Creatine kinase and calcium plasma levels and renal function parameters of control and experimental groups, 4 days after saline or glycerol injections.

	Control	Control + Calcitriol	Glycerol	Glycerol + Calcitriol
CK	26.79 ± 1.59	24.57 ± 4.89	44.23 ± 7.51*^,+^	45.42±3.13*^,+^
Ca^2+^	10.04 ± 0.50	9.507 ± 0.41	8.144 ± 0.50	8.373 ± 0.19
Cr	0.610 ± 0.03	0.620 ± 0.05	2.73 ± 0.39*^,+^	2.07±0.43*^,+^
GFR	0.421 ± 0.06	0.490 ± 0.03	0.113 ± 0.02*^,+^	0.240 ± 0.04*^,+,#^
FE_Na+_ (%)	0.290 ± 0.06	0.317 ± 0.05	3.713±0.51*^,+^	1.96±0.64^+,#^
U_osm_	2080±241.6	1809±117.9	541.5±25.49*^,+^	696.0±33.45*^,+^
V	0.004 (0.002;0.01)	0.005 (0.003;0.01)	0.020 (0.01;0.02)*^,+^	0.013 (0.007;0.01)*^,+,#^

CK, plasma creatine kinase (U/L); Ca^2+^, plasma calcium (mg/dL); Cr, plasma creatinine (mg%); GFR, glomerular filtration rate (ml min^−1^100 g^−1^); FE_Na+_, fractional sodium excretion (%); Uosm, urinary osmolality (mOsm kg H_2_O^−1^); V, urinary flow (mL/min). The data are expressed as the mean ± SEM (GFR, FE_Na+_ and Uosm) or median and interquartile range (25–75%). n = 7–12 per group. *P < 0.05 compared to Control; ^+^*P* < 0.05 compared to Control ^+^Calcitriol; ^#^*P* < 0.05 compared to Glycerol.

### Light microscopy and morphometric studies

**Figure 1:**
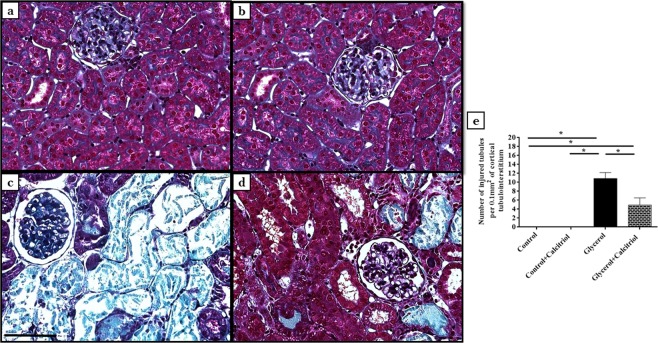
Histological sections stained with Masson’s Trichrome representative of control (**a**), control + calcitriol (**b**), glycerol (**c**) and glycerol + calcitriol (**d**) animals. Number of injured tubules (**e**) of the different groups. Note that tubulointerstitial lesions are more intense in (**c**) than in (**d**). The bar indicates 50 μm. The data are expressed as the means ± SEM. *Denotes a statistical significance of *P* < 0.05 between the groups. (n = 7–10 per group). Magnification, ×400.

In animals of both groups treated with glycerol, the histological studies revealed the presence of acute tubular necrosis (ATN), characterized by necrotic cells in the tubular lumen, loss of the brush border of tubule cells and increases in the tubular lumen 4 days after glycerol injection (Fig. 1a–d). The quantitative evaluation of the tubulointerstitial involvement of the renal cortex showed that the number of injured tubules (Fig. 1e) was significantly higher in animals in the glycerol group than in the controls and the animals from the group treated with glycerol + calcitriol.

### Immunohistochemical studies

**Figure 2:**
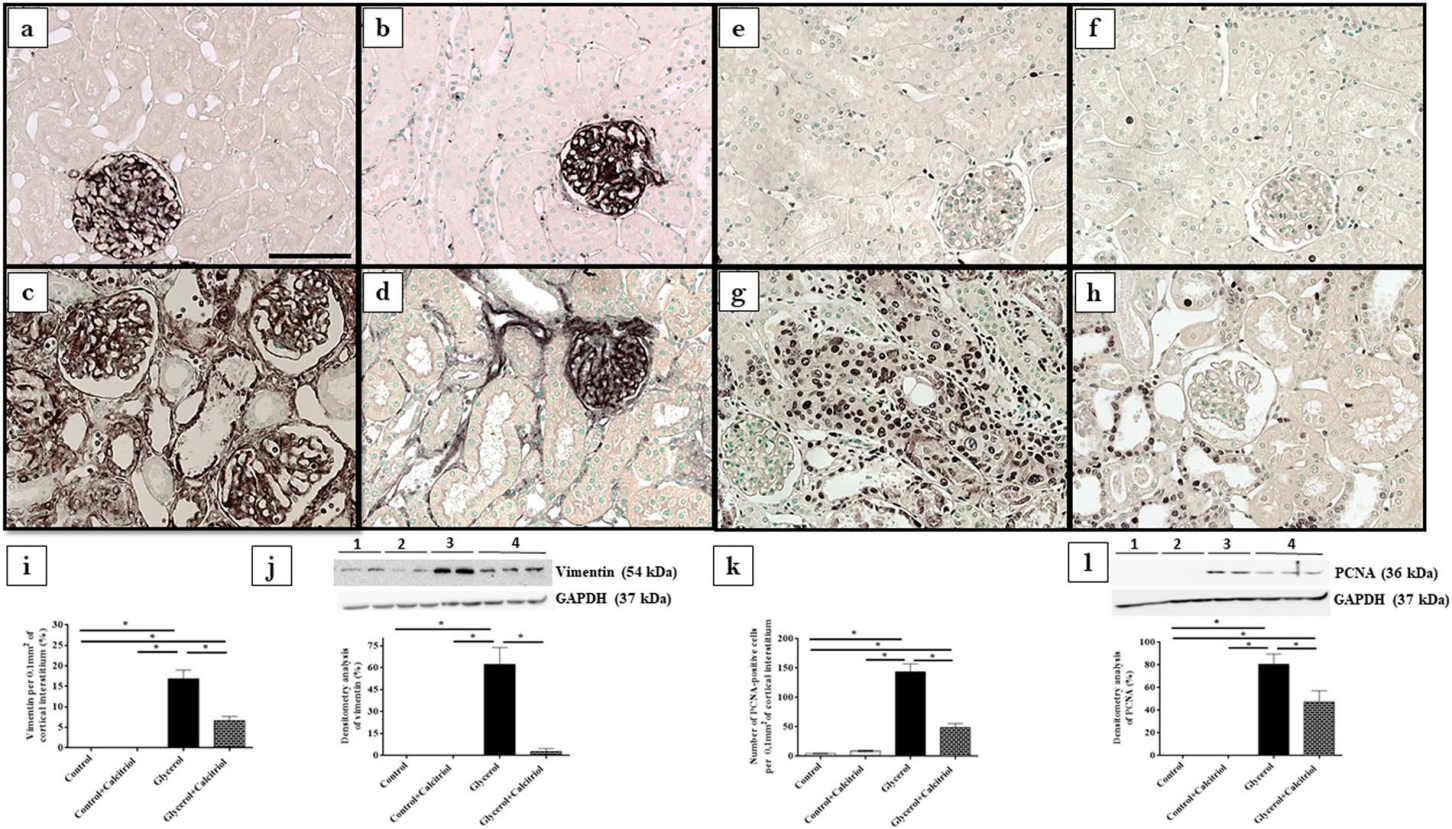
Immunolocalization of vimentin (**a**–**d**) and PCNA (**e**–**h**) in the renal cortex of control (**a**,**e**), control + calcitriol (**b**,**f**), glycerol (**c**,**g**) and glycerol + calcitriol (**d**,**h**) animals. The bar indicates 50 μm. Percentage of tubulointerstitial area in marked with vimentin (**i**) and number of PCNA^+^ cells (**k**) in the renal cortex. Western blot analysis of vimentin (**j**) and PCNA (**l**) in the renal cortex of control (lane 1), control + calcitriol (lane 2), glycerol (lane 3), and glycerol + calcitriol (lane 4) animals. The densitometric ratio between vimentin or PCNA and GAPDH was calculated, and the data are expressed compared with those of the control group, with the mean (±SEM) control value designated 100% and expressed as the means ± SEM. Blots are representative imagens from independent experiments. *Denotes a statistical significance of *P* < 0.05 between the groups. (n = 6–7 for each group). Magnification, ×400.

**Figure 3:**
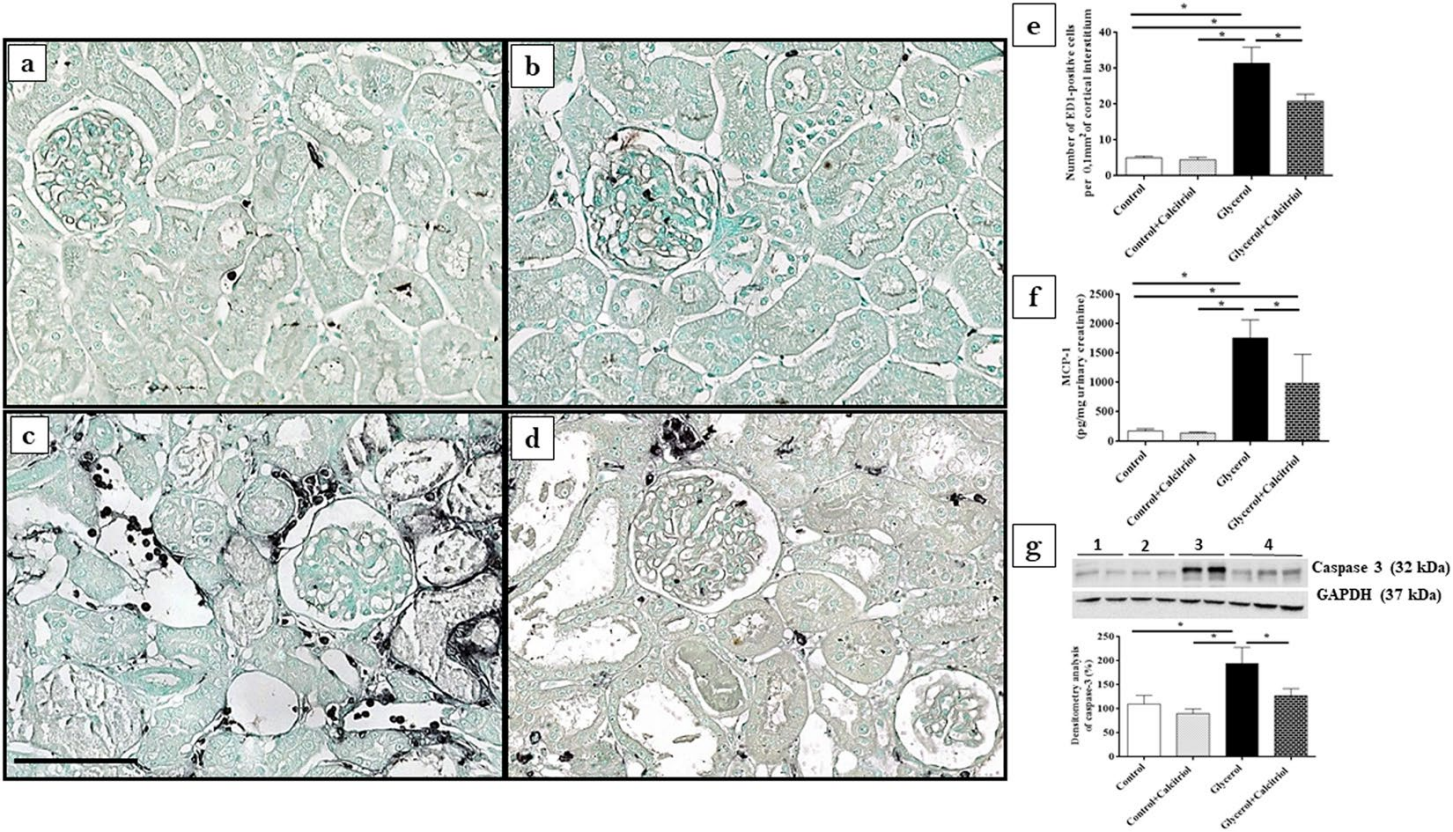
Immunolocalization of ED1^+^ cells (macrophages) in the renal cortex of control (**a**), control + calcitriol (**b**), glycerol (**c**) and glycerol + calcitriol (**d**) animals and number of ED1+ cells in the tubulointerstitial compartment (**e**). The bar indicates 50 μm. Levels of urinary MCP-1 (**f**) evaluated by ELISA of animals from different groups. Western blot analysis of caspase 3 (**g**) in the renal cortex of control (lane 1), control + calcitriol (lane 2), glycerol (lane 3) and glycerol + calcitriol (lane 4) animals. The densitometric ratio between caspase 3 and GAPDH was calculated, and the data are expressed compared with that of the control group, with the mean (±SEM) control value designated 100%. Blots are representative images from independent experiments. The data are expressed as the means ± SEM. *Denotes a statistical significance of *P* < 0.05 between the groups. (n = 7–12 per group). Magnification, ×400.

**Figure 4:**
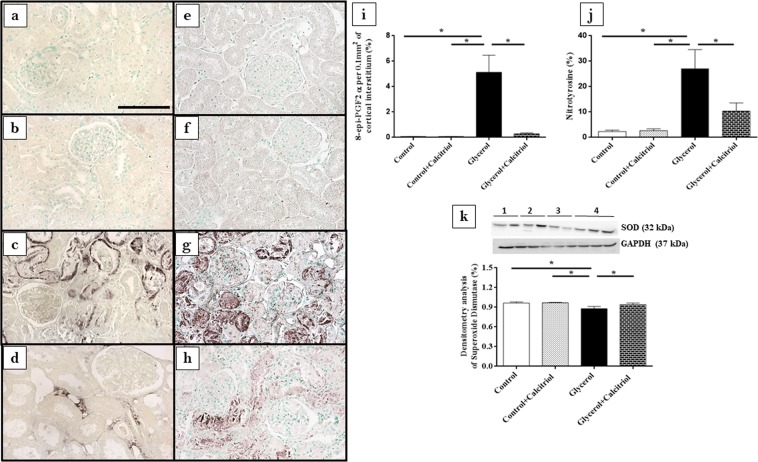
Immunolocalization of 8-epi-PGF2 α (**a**–**d**) and nitrotyrosine (**e**–**h**) in the renal cortex of control (**a**,**e**), control + calcitriol (**b**,**f**), glycerol (**c**,**g**) and glycerol + calcitriol (**d**,**h**) animals. The bar indicates 50 μm. Percentage of the tubulointerstitial area of the renal cortex marked with 8-epi-PGF2 α (**i**) and nitrotyrosine (**j**) in the different groups. The data are expressed as the means ± SEM. Western blot analysis of superoxide dismutase (**k**) in the renal cortex of control (lane 1), control + calcitriol (lane 2), glycerol (lane 3) and glycerol + calcitriol (lane 4) animals. The densitometric ratio between superoxide dismutase and GAPDH was calculated, and the data are expressed compared with that of the control group, with the mean (±SEM) control value designated 100%. Blots are representative imagens from independent experiments. The data are expressed as the means ± SEM. *Denotes a statistical significance of *P* < 0.05 between the groups. (n = 6–7 for each group). Magnification, ×400.

**Figure 5:**
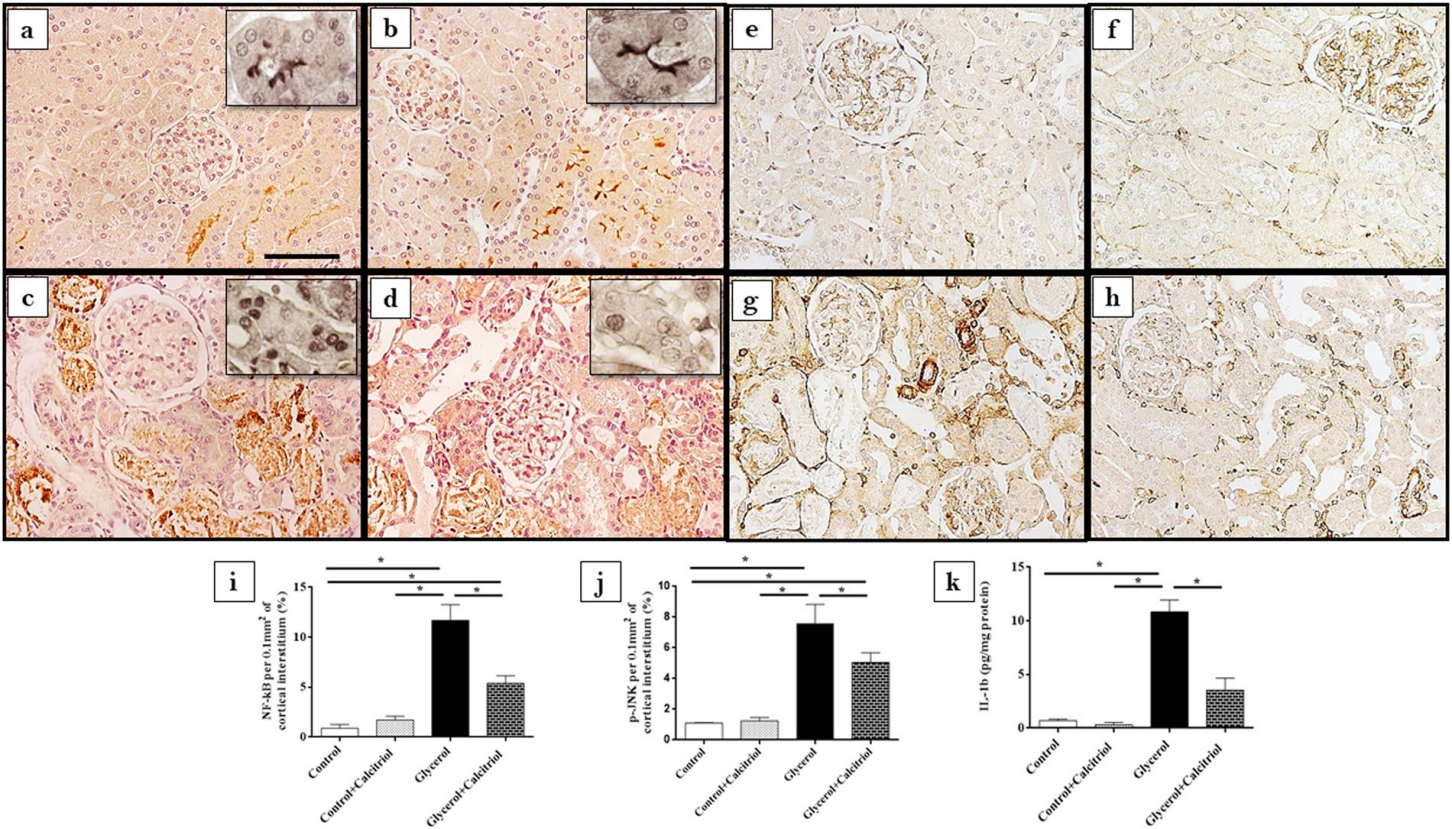
Immunolocalization of NF-κB (**a**–**d**) and p-JNK (**e**–**h**) in the renal cortex of control (**a**,**e**), control + calcitriol (**b**,**f**), glycerol (**c**,**g**) and glycerol + calcitriol (**d**,**h**) animals. The bar indicates 50 μm. Inset: slices without counterstaining. Percentage of the tubulointerstitial area of the renal cortex marked with NF-κB (**i**) and p-JNK (**j**) in the animals from different groups. Levels of IL-1β in the renal cortex (**k**) evaluated by ELISA of animals from different groups. The data are expressed as the means ± SEM. *Denotes a statistical significance of *P* < 0.05 between the groups. (n = 7–12 per group). Magnification, ×400; inset magnification, ×840.

**Figure 6:**
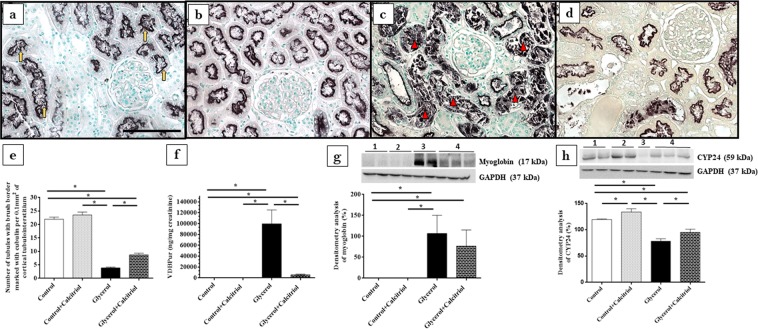
Immunolocalization of cubilin in the renal cortex of control (**a**), control + calcitriol (**b**), glycerol (**c**) and glycerol + calcitriol (**d**) animals. The bar indicates 50 μm. Number of tubules with brush border marked with cubilin of animals from different groups (**e**). Note that labeling for cubilin is present in the brush border of the tubular cell in controls animals (yellow arrow), whereas in animals of the glycerol group the labeling is more present in the tubular lumen (red arrowhead) due to loss of the epithelial cells from the proximal tubule, as a consequence of acute tubular injury; this commitment is lower in animals of the glycerol + calcitriol group. Urinary excretion of VDBP evaluated by ELISA in the different groups (**f**). Western blot analysis of myoglobin expression (**g**) and CYP24 (**h**) in the renal cortex of control (lane 1), control + calcitriol (lane 2), glycerol (lane 3), and glycerol + calcitriol (lane 4) animals. The densitometric ratio between myoglobin, CYP24 and GAPDH was calculated, and the data are expressed compared with that of the control group, with the mean (±SEM) control value designated 100%. Blots are representative imagens from independent experiments. The data are expressed as the means ± SEM. *Denotes a statistical significance of *P* < 0.05 between the groups. (n = 6–7 for each group). Magnification, ×400.

Immunohistochemical analysis showed increased expression of vimentin (Fig. 2a–d,i) and PCNA-positive cells (PCNA+) (Fig. 2e–h,k) in the tubule cells from the renal cortex of the animals from the glycerol group. We also observed increased inflammatory cell infiltrates, as evidenced by the higher number of cells positive for ED1 (ED1+, macrophages) (Fig. 3a–e). In the kidneys of these animals, increased 8-epi-PGF2 α (Fig. 4a–d,i) and nitrotyrosine (Fig. 4e–h,j) expression levels, markers of oxidative damage, were also observed. The NF-κB (Fig. 5a–d,i) and p-JNK (Fig. 5e–h,j) expression levels were also higher in the nucleus from the cortical tubule interstitium and in the tubule cells of the kidney from the glycerol-treated animals, confirming the presence of an inflammatory process. Treatment with calcitriol reduced all these alterations induced by glycerol. We also observed that the number of tubules with a brush border marked with cubilin was smaller in the glycerol-treated than in glycerol + calcitriol-treated animals (Fig. 6a–e).

### Western blot studies

The results of Western blot analyses for vimentin (Fig. 2j) and PCNA (Fig. 2l) confirmed the results found in the immunohistochemical analyses for these proteins. There was higher expression of both in the renal cortex of the animals in the glycerol group than in the control and glycerol + calcitriol groups. The increase in caspase 3 (Fig. 3g) expression in the glycerol group animals was prevent by treatment with calcitriol. The decreased expression of EC-SOD (Fig. 4k) and CYP24 (Fig. 6h) in the glycerol group was also attenuated in the glycerol + calcitriol group. However, expression levels of myoglobin in the renal cortex were higher in both glycerol-treated animals (Fig. 6g) than in the respective controls.

### ELISA studies

Analysis of IL-1β expression in renal tissue showed higher levels of this cytokine in the renal cortex in the glycerol group than in the controls (Fig. 3g), which was attenuated by calcitriol treatment. There was greater urinary excretion of MCP-1 (Fig. 3f) and VDBP (Fig. 6f) in glycerol-treated animals than in the controls. These increases were less intense in the animals in the glycerol + calcitriol group.

## Discussion

Glycerol-induced rhabdomyolysis was evidenced by increased plasma creatine kinase concentrations compared to control animals. These animals had alterations in renal function characterized by increased fractional excretion of sodium and urinary volume, as well as decreased GFR and urinary osmolality. Treatment with calcitriol attenuated the increases in urinary volume and fraction excretion of sodium and the decrease in GFR caused by glycerol injection. There was no difference in the calcium plasma levels at day four after the injury induced by glycerol. However, it has been already observed muscle regeneration at the third day after the injury in this model and the lesion is less intense at this time^23^.

Morphological data showed that structural damage such as injury tubular in the renal cortex in the animals injected with glycerol was also less intense in animals treated with calcitriol.

Tubular cell lesions were also shown by the increased expression of vimentin in the renal cortex from glycerol injected rats. Tubular cells only express vimentin when they are proliferating, showing recent lesions of these cells^24,25^. This result was confirmed by the increased number of PCNA-positive cells. Calcitriol treatment reduced cell tubular injury and the expression of vimentin and PCNA in the animals injected with glycerol. Tan *et al*.^20^ observed that the treatment with a synthetic vitamin D analogue reduced the expression of PCNA and attenuated the renal interstitial fibrosis in a model of obstructive nephropathy. The authors also observed that vitamin D treatment restored the expression of VDR receptor, blocked the epithelial-mesenchymal transition and inhibited cell proliferation, demonstrating that vitamin D plays a protective role in cellular integrity against this cell injury process. In the present study, we observed that the lesions in the renal cortex were also associated with decreased cubulin expression in the apical region of the tubule cells and with increased urinary excretion of VDBP in animals in the glycerol group. The reduction in the number of tubules expressing cubilin in the cell brush border could lead to disturbances in vitamin D activation. This is also demonstrated by the reduction of CYP24 expression in animals of the glycerol group. CYP24 is strongly induced by calcitriol and can be used as a marker of 1,25(OH)2D effect in that cell^26^. The organism tends to compensate for the lack of calcitriol 152 by reducing the expression of CYP24, which is involved in its metabolism.

Published data suggest that vitamin D deficiency is associated with the severity of AKI^27^, with activation of proinflammatory pathways^28^ aggravating tubulointerstitial damage and fibrosis. In the present study, calcitriol-treated rats presented largely preserved cubilin receptors, demonstrating the renoprotective role of calcitriol. In addition, studies by Luchi *et al*.^29^ showed that vitamin D deficiency is a risk factor for contrast-induced AKI due to imbalance in intrarenal vasoactive substances and oxidative stress. Our results showed that increased renal expression of 8-epi-PGF2α, (isoprostane, marker of oxidative damage) as well as that of nitrotyrosine (a marker of protein nitration, a consequence of oxidative damage) and the decreased expression of EC-SOD (an antioxidant enzyme) induced by glycerol were attenuated by calcitriol treatment. Both glycerol-treated groups had increased macrophage numbers, NF-κB, caspase 3 and p-JNK expression levels, IL-1β levels in renal tissue and of MCP-1 in urine, all of which were attenuated by treatment with calcitriol, showing reduction of the inflammatory effect induced by glycerol. It is important to note that isoprostanes, products of the oxidation of arachidonic acid^30^, are also potent vasoconstrictors that could contribute to the fall in GFR observed in the present study. The NF-κB and p-JNK pathways can be activated by oxidative stress and glycerol-induced inflammatory processes^6,13,31^. The glycerol + calcitriol group showed predominantly cytoplasmic NF-κB labelling, whereas in the glycerol group, labelling was unclear. Using the AKI model induced by lipopolysaccharide, Xu *et al*.^32^ showed that in animals treated with vitamin D, activated vitamin D receptor (VDR) inhibited the renal NF-κB pathway through its physical interaction with the p65 subunit of NF-κB^32^. Therefore, the interaction between renal VDR and the p65 subunit might be responsible, at least in part, for the calcitriol-mediated anti-inflammatory activity in this model.

The present study showed that the expression of myoglobin in renal tissue was increased in both glycerol-treated groups, and calcitriol treatment did not prevent this accumulation. Therefore, calcitriol should act directly on the attenuation of the oxidative damage and inflammatory processes.

**Figure 7:**
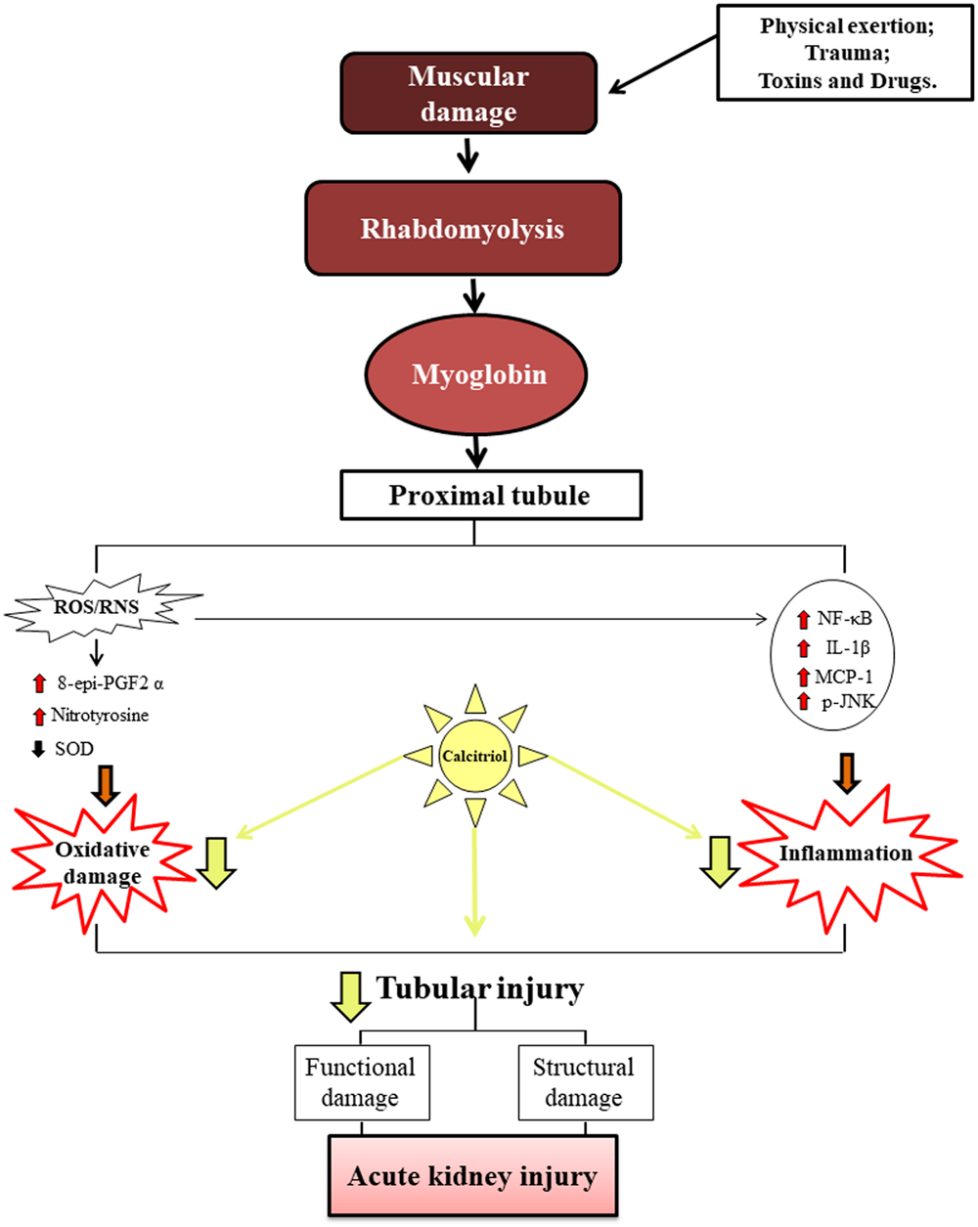
Rhabdomyolysis has been associated with different conditions, including severe trauma, intense physical exercise, toxins and drugs. Regardless of its cause, myoglobin-induced renal toxicity by several factors: hypovolemia, nephrotoxicity in proximal tubule cells and myoglobin precipitation in distal tubule leading to tubular obstruction. As a consequence, increased oxidative damage and inflammation is observed in renal tissue with loss of renal function. All these damages were reduced with the treatment with calcitriol, this having an important role antioxidant and anti-inflammatory.

In summary, the results obtained in the present study showed that calcitriol had a protective effect on renal damage induced by glycerol, attenuating renal functional and structural alterations, as well as the inflammatory processes and oxidative damage (Fig. 7). These effects were not associated with decreased myoglobin expression in renal tissues.

## Material and Methods

### Animals and experimental protocols

Male Wistar Hannover rats (weighing 180–200 g) were provided by the Animal House of the Ribeirão Preto University of São Paulo, Brazil and were maintained in groups of five per cage in a 12-h-12-h dark-light cycle under standard environmental conditions (22 °C), with water and chow provided *ad libitum*.

AKI was induced by intramuscular (IM) injection of 50% glycerol (8 ml/kg; Sigma Chemical Company, St. Louis, USA) diluted in saline (0.9% NaCl) into the hind limbs. Control animals received vehicle (saline) injection via the same route. Some animals received calcitriol (6 ng/day; Abbott, Chicago, USA); the others received vehicle via mini-osmotic pumps (Model 2004, Alzet, Cupertino, CA) implanted subcutaneously (SC) into the back of the animals under anaesthesia with isoflurane. This calcitriol dose was selected according to Kuhlmann *et al*. studies^33^ that showed the beneficial effect of calcitriol in a subtotal nephrectomy model and in a previous study from our laboratory^19^. Treatment with calcitriol started three days before the administration of glycerol or vehicle and continued until the fourth day after the injections. Rats had water restriction 24 h before the injection of glycerol or saline and 1 h after administration. The animals were divided into four groups: (a) control (n = 7), receiving vehicle SC and injection of saline IM; (b) control + calcitriol (n = 7), receiving calcitriol SC and injection of saline IM; (c) glycerol (n = 13), receiving vehicle SC and IM glycerol injection; and (d) glycerol + calcitriol (n = 10), receiving calcitriol SC and injection of glycerol IM.

The 24-h urine samples were collected in metabolic cages, 3 days after saline or glycerol injections, and their volumes were measured. On day 4 after the injections, the rats were anaesthetized intraperitoneally using sodium thiopental (40 mg/kg), and the aorta was cannulated for the collection of blood samples to renal function and biochemical studies. The left kidney was removed, transversely sectioned and fixed in methacarn solution (methanol, chloroform, and acetic acid), rinsed in 70% ethanol and processed for paraffin embedding for histological and immunohistochemical study. The right kidney was perfused through the aorta with phosphate-buffered saline (PBS; 0.15 M NaCl and 0.01 sodium phosphate buffer, pH = 7.4) for Western blot and ELISA studies.

All experimental procedures were conducted in accordance with the principles and procedures outlined in the National Institutes of Health (NIH) Guide for the Care and Use of Laboratory. The animal experiments were approved by the Committee of the University of São Paulo at Ribeirão Preto School of Medicine (protocol no. 186/2016).

### Renal function and plasma calcium and creatine kinase levels

The 24-h urine and plasma samples were used for creatinine and sodium quantification for the evaluation of the glomerular filtration rate (GFR) and sodium excretion fraction. Plasma and urinary creatinine levels were determined using a commercial kit (Labtest, Lagoa Santa, Brazil). Urinary and plasma sodium levels were analysed using a quantitative electrode quantification technique (9180 Electrolyte Analyzer, Roche Diagnostics GmbH, Mannheim, Germany). Urinary osmolality was determined by the freezing method (The Advanced Osmometer, Model 3250; Advanced Instruments, Norwood, USA). Plasma calcium and creatine kinase were determined using a commercial kit (Labtest, Lagoa Santa, Brazil and Laborlab, São Paulo, Brazil respectively).

### Light microscopy and morphometric studies

Histological sections (3-μm) were stained with Masson’s Trichrome and examined under light microscopy. The tubulointerstitial lesions were evaluated by determining the number of injured tubules at 30 fields/kidney (measuring 0.1 mm^2^) from the renal cortex, and the mean values were calculated per kidney.

### Immunohistochemical studies

Histological sections were deparaffinized using xylol and subjected to immunohistochemical studies. The sections were incubated with either monoclonal anti-p-JNK (1/30; Santa Cruz Biotechnology, CA, USA), polyclonal anti-nuclear factor kappa B (NF-κB) (1/80; Santa Cruz Biotechnology, CA, USA), polyclonal anti-8-epi-prostaglandin F2α (8-epi-PGF2 α) (1/500; Oxford Biomedical Research, Oxford, UK), or polyclonal anti-nitrotyrosine (1/1000; Upstate, CA, USA) antibodies at 4 °C overnight or with monoclonal anti-ED1 (1/1000; Serotec, Oxford, UK), monoclonal anti-proliferative cell nuclear antigen (PCNA) (1/1000; Sigma Chemical Company, St Louis, USA), monoclonal anti-vimentin (1/500; Dako, Glostrup, Denmark) or polyclonal anti-cubilin (1/1500; Santa Cruz Biotechnology, CA, USA) antibodies for 1 h at room temperature. The reaction products were detected using an avidin-biotin-peroxidase complex (Vector Laboratories, Burlingame, USA), and the colour was developed by incubation with 3,3′-diaminobenzidine (DAB; Sigma Chemical Company, St. Louis, USA) and nickel chloride in the presence of H_2_O_2_. Counter-staining was performed with methyl green or haematoxylin. We also performed immunohistochemical studies for NF-κB using slices without counter-staining to check for the presence of nuclear staining. Next, the material was dehydrated and mounted, and then non-specific binding was blocked by diluting the primary and secondary antibodies in a PBS solution containing 1% bovine serum albumin (BSA) (Sigma Chemical Company, St. Louis, USA).

The evaluation of immunoperoxidase staining for ED1 and PCNA was performed by counting the positive cells of the renal cortical tubulointerstitium. The reactions for vimentin, 8-epi-PGF2 α, nitrotyrosine, NF-κB and p-JNK were evaluated by analysing the percentage of the renal cortical tubulointerstitium (0.1 mm^2^) using NIH ImageJ software (http://www.nih.gov). The reaction for cubilin was evaluated by counting the number of tubules with a brush border marked with cubilin in the renal cortical tubules. We evaluated 30 consecutive fields of the renal cortical tubules or tubulointerstitium (measuring 0.1 mm^2^) of each animal and determined, for each rat, the number of ED-1 or PCNA-positive cells, the mean percentage of the marked area with vimentin, 8-epi-PGF2 α, nitrotyrosine, NF-κB or p-JNK, or number of marked tubules with cubilin per field.

### Western blot studies

The samples were homogenized with lysis buffer [Tris-HCl (50 mM, pH 7.4), NaCl (150 mM), Triton X-100 (1%), sodium dodecyl sulphate (SDS; 1 μg/ml), leupeptin (1 μg/ml), phenylmethylsulfonyl fluoride (1 mM), sodium orthovanadate (1 mM, pH 10), sodium pyrophosphate (1 μg/1 mM), sodium fluoride (25 mM), ethylenediamine tetraacetic acid (EDTA; 0.001 M, pH 8)] and centrifuged at 4 °C for 15 minutes at 10000 rpm. The proteins were separated by polyacrylamide gel electrophoresis, transferred to nitrocellulose membranes, incubated for 1 h in 30 ml of blocking buffer (tris buffered saline [TBS]; 5% skim milk), washed in buffer (TBS, 0.1% Tween 20, pH 7.6) and incubated with anti-vimentin (1/1500; Dako, Glostrup, Denmark), anti-PCNA (1/1000; Sigma Chemical Company, St Louis, USA), anti-EC-SOD (extracellular superoxide dismutase, an antioxidant enzyme, 1/500; Assay Designs, Ann Arbor, USA), anti-caspase 3 (1/100; Santa Cruz Biotechnology, CA, USA), anti-CYP24 (1/500; Abnova, CA, USA) or anti-myoglobin (1/200; Santa Cruz Biotechnology, CA, USA) overnight at 4 °C. To adjust the equivalence of protein loading and/or transfer, the membranes were also incubated with anti-glyceraldehyde-3-phosphate dehydrogenase (GAPDH) antibody (1/1000; Cell Signaling Technology, Danvers, EUA) overnight at 4 °C. The blots were then washed and incubated with horseradish peroxidase-conjugated goat anti-mouse (1/10,000; Dako, Glostrup, Denmark) for 1 h at room temperature. The membrane-bound antibodies were detected with the Supersignal West Pico Chemiluminescent substrate (Pierce, Rockford, IL, USA) and were captured on X-ray film. The intensity of the identified lanes was quantified by densitometry with ImageJ NIH image software (http://www.nih.gov) and was reported in arbitrary units. Protein estimations were performed using the Bradford method^34^.

### ELISA studies

The levels of VDBP (vitamin D binding protein) and MCP-1 (macrophage chemoattractive protein-1) were measured in urine samples, and the levels of IL-1β were measured in renal tissue samples. The VDBP and MCP-1 content were quantified in urine samples collected from the urinary bladder treated with 1 mM phenylmethylsulfonyl fluoride (PMSF; Sigma Chemical Co, St. Louis, Missouri, USA), and then the samples were stored at −70 °C until analysis. The contents were determined using ELISA kits according to the manufacturer’s guidelines (Alpco, Keewaydin Drive, Salem, USA; Pierce, Rockford, USA; and R&D Systems Inc., Minneapolis, USA, respectively). The values of VDBP and MCP-1 were expressed as ng/mg of creatinine and IL-1β as pg/mg of protein.

### Statistical analysis

Two-way analysis of variance (ANOVA) with the Newman-Keuls multiple comparison test was used for the normally distributed variables or those that showed a normal distribution after log_e_ transformation. The normality of the dependent variables was investigated using the graphics of normality and dispersion and by the Kolmogorov-Smirnov test. Statistical analyses were performed using the Statistica program version 10 (StatSoft, Tulsa, USA). Graphics were constructed using GraphPad Prism version 6.0 for Windows (GraphPad Software, La Jolla, USA). The level of statistical significance was set at p < 0.05.

## Supplementary information

Suplementary Info

## Data Availability

The datasets generated during the current study are available from the corresponding author on reasonable request.
