# Supplementary material for: Protective effect of calcitriol on rhabdomyolysis-induced acute kidney injury in rats
Source: Sci Rep. 2019 May 8;9:7090. doi: 10.1038/s41598-019-43564-1 (PMC6506495; doi:10.1038/s41598-019-43564-1)

Protective effect of calcitriol on rhabdomyolysis-induced acute kidney injury in rats

Natany Garcia Reis[1], Heloísa Della Coletta Francescato[1], Lucas Ferreira de Almeida[1], Cleonice Giovanini Alves da Silva[1], Roberto Silva Costa[2], Terezila Machado Coimbra[1,*]

Figure 2J

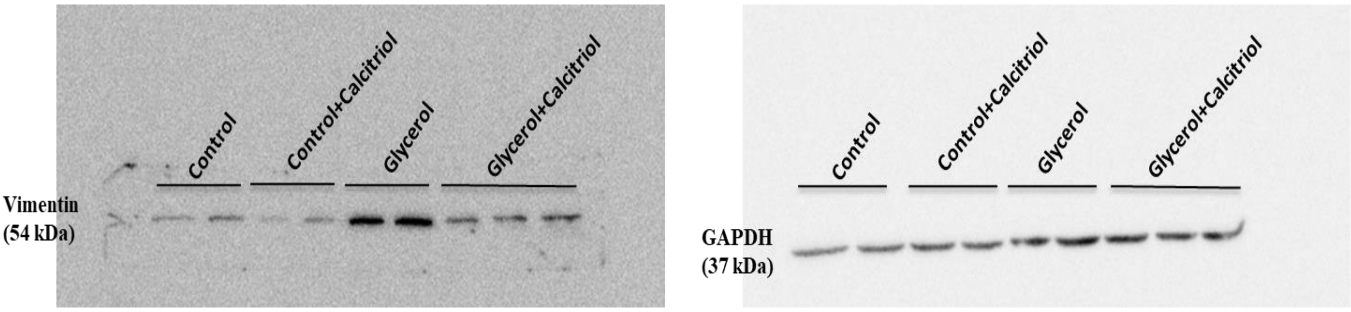

Figure 2L

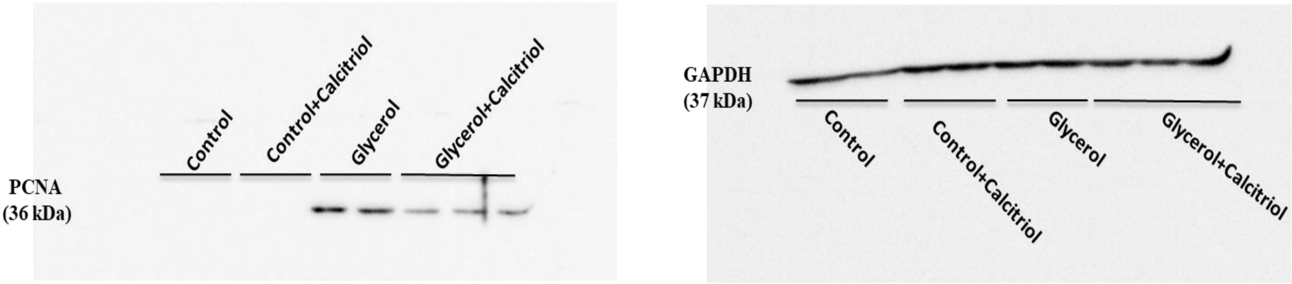

Figure 3G

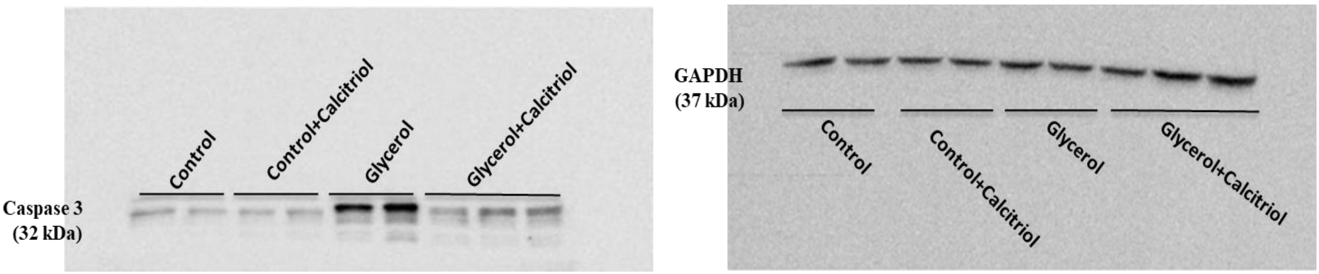

Figure 4K

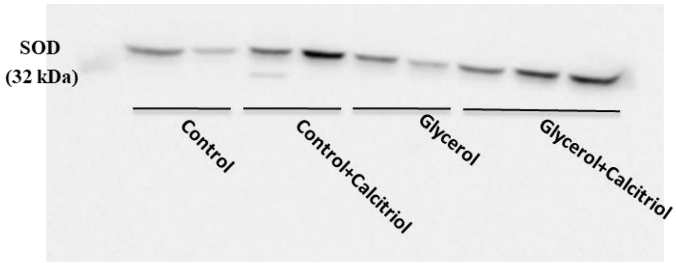

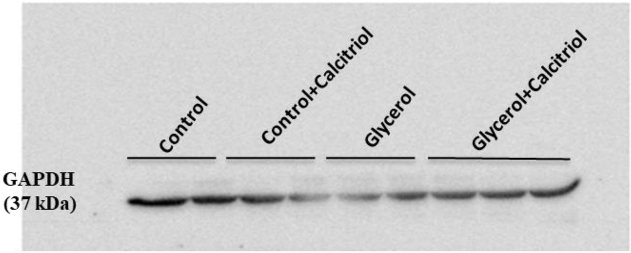

Figure 6G

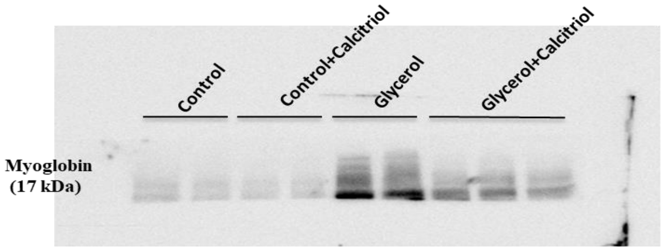

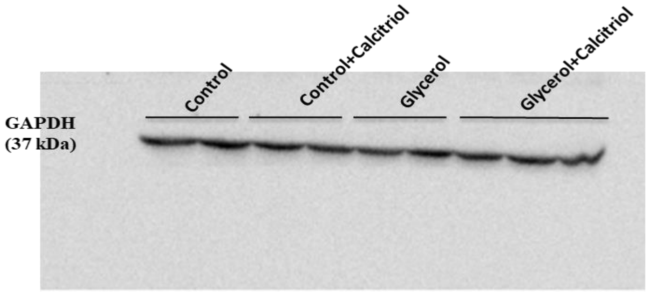

Figure 6H

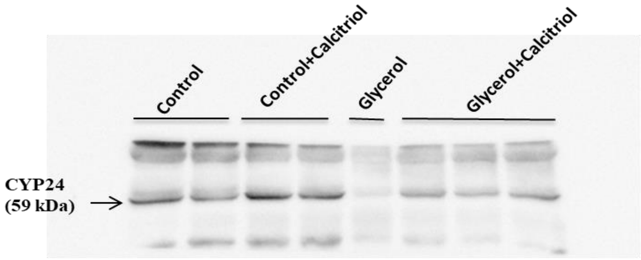

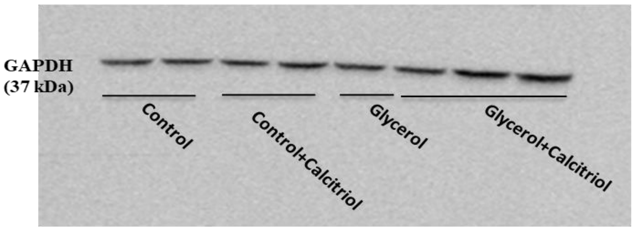

Supplement: Supplementary file 1 — Suplementary Info [file 41598_2019_43564_MOESM1_ESM.pdf]
